# Eicosapentaenoic acid and branched-chain amino acids fortified complete nutrition drink improved muscle strength in older individuals with inadequate protein intake

**DOI:** 10.3389/fnut.2023.1164469

**Published:** 2023-06-30

**Authors:** Watcharapol Khoonin, Prapimporn Chattranukulchai Shantavasinkul, Chalat Santivarangkna, Kemika Praengam, Dunyaporn Trachootham

**Affiliations:** ^1^Doctor of Philosophy Program in Nutrition, Faculty of Medicine, Ramathibodi Hospital and Institute of Nutrition, Mahidol University, Bangkok, Thailand; ^2^Faculty of Medicine, Ramathibodi Hospital, Mahidol University, Bangkok, Thailand; ^3^Institute of Nutrition, Mahidol University, Nakhon Pathom, Thailand

**Keywords:** EPA, BCAA, sarcopenia, muscle, complete nutrition drink, elderly, protein, randomized controlled trial

## Abstract

**Background:**

Elevated inflammation and negative nutritional balance contribute to sarcopenia, a progressive loss of muscle mass, strength, and function. This study investigated the effect of energy supplementation and the combination of anti-inflammatory factor (eicosapentaenoic acid; EPA) and muscle-synthesis promotor (branched-chain amino acids; BCAA) on body composition, muscle, and inflammatory biomarkers in elderly with inadequate protein intake.

**Methods:**

A randomized blinded placebo-controlled trial was conducted on 84 elderly with inadequate protein intake. The participants were randomly assigned into four groups receiving a complete nutrition drink; (1) control formula, (2) fortified with 2.2 g EPA, (3) with 2.2 g EPA and 5 g BCAA (2:1:1 of Leu: Ile: Val), and (4) with 2.2 g EPA plus 5g BCAA (4:1:1 of Leu: Ile: Val). Each subject consumed two sachets of the drink to gain 500 kcal/day and performed arm muscle exercises for 3 weeks. Body compositions and handgrip strength were measured using BIA and a dynamometer, respectively. Plasma EPA and BCAA levels were determined using LC-MS/MS to ensure compliance. Muscle protein biomarkers including histidine, β-alanine, and carnosine were measured using LC-MS/MS. Serum inflammatory (IL-6) and anti-inflammatory cytokines (IL-10) were measured by using ELISA.

**Results:**

No symptoms and signs of adverse events were observed. The right arm muscle mass and handgrip strength were significantly increased after consuming a complete nutrition drink fortified with EPA + BCAA 2:1:1 and 4:1:1 of Leu: Ile: Val (*p* < 0.05 and *p* < 0.01, respectively. Consistently, consuming such combinatory formula non-significantly elevated carnosine with reduced histidine, and increased IL-10 with decreased IL-6. All relevant intervention groups showed a significant increase in plasma levels of BCAA and EPA.

**Conclusion:**

Consuming a complete nutrition drink fortified with 2.2g EPA and 5g BCAA 2:1:1 or 4:1:1 of Leu: Ile: Val for 3 weeks may increase right arm muscle mass and strength in elderly with inadequate protein intake. The tendency of increased dipeptide (carnosine)/decreased free amino acid (histidine) suggests a shift toward muscle protein synthesis. The trend of decreased inflammatory/increased anti-inflammatory cytokines suggests an anti-inflammatory effect. Future long-term studies are warranted to confirm the combinatory effect of BCAA and EPA in the prevention of sarcopenia.

**Clinical trial registration:**

Thailand Clinical Trial Registry No. TCTR20230116005.

## Introduction

Older populations are at risk of sarcopenia, i.e., an age-related, involuntary loss of skeletal muscle mass, and strength (1). Compelling evidence suggests that skeletal muscle mass and skeletal muscle strength drop linearly, with up to 50% loss of muscle mass by the age of 80 and above (1). Protein-energy malnutrition is associated with an increased risk of sarcopenia (2, 3). Our and other previous studies showed that inadequate protein intake is associated with low muscle mass index in elderly people (4, 5). Worsen the problem, aging leads to the deterioration of organs involved in nutrition intake and metabolism; thus, the risk of malnutrition-related sarcopenia is high in the elderly population (6–8). Importantly, sarcopenia can reduce the quality of life, lessen tolerance to illness, and increase the risk of mortality (9, 10). Nevertheless, no standard interventions for sarcopenia had been accepted, and neither does conventional nutritional support can exclusively resolve sarcopenia (11). The European Society for Clinical Nutrition and Metabolism (ESPEN) recommends using a multimodal approach in the management of sarcopenia (12). Convincing pieces of evidence suggested that the mechanism of sarcopenia involves systemic inflammation leading to increased degradation of protein and lipid from muscle and adipose tissue storage (13).

Previous studies demonstrated the potential benefit of eicosapentaenoic acid (EPA) to alleviate sarcopenia in the elderly, showing improvement in body weight, muscle mass, and function (14). A possible mechanism of EPA likely involves anti-inflammation and downregulation of proteasome expression (15). The anti-inflammatory mechanisms of EPA include protection against the disruption of the cell membrane and inactivation of transcription factor NFκB leading to the suppression of gene transcription for pro-inflammatory cytokines such as IL-1, IL-6, IL-8, and IL-12 (16, 17). Furthermore, EPA is also an inhibitor of platelet-activating factor (PAF) (18), which is a potent lipid inflammatory mediator (19) negatively affecting whole-body and skeletal muscle protein synthesis (20) Moreover, a plasma lipid profile of lower omega 6, which antagonize omega 3, has been associated with reduced PAF biosynthesis and/or increased catabolism (21). A dietary pattern rich in fish and other healthy food groups has been inversely associated with circulating PAF plasma levels (22). Interestingly, a recent study showed that the supplementation of an amino acid-rich diet after exercise could preserve lean body mass in older persons (23). Another study showed that a single dose of amino acid-rich complete nutrition drinks could stimulate the rate of muscle synthesis (24). The effect may be due to branched-chain amino acids (BCAAs) especially leucine which is critical protein synthesis for muscle (24). Leucine was shown to be the primary stimulator of muscle protein synthesis via the mammalian target of the rapamycin complex 1 (mTORC1) pathway (25). Therefore, the elevation of plasma leucine concentration by providing leucine-enriched nutrition supplements with an adequate energy drink can resume muscle protein synthesis, while appropriate menu changes can increase leucine dietary intake in patients (26). Nevertheless, it is still unclear if a combination between EPA and BCAA would be effective in improving muscle health and reducing the risk of sarcopenia in the elderly at risk. Recently, the EPA–BCAA-fortified nutrition drink has been developed and sterilized with a retort. It passed the microbial safety test and had over 80% sensory acceptance (27). Thus, this study aimed to investigate the effect of energy supplementation and the combination of anti-inflammatory factor (eicosapentaenoic acid; EPA) and muscle-synthesis substrate (branched-chain amino acids; BCAA) on body composition, muscle degradation, and inflammatory biomarkers in elderly people with inadequate protein intake.

## Materials and methods

### Ethical aspects and setting

Data were collected at the Institute of Nutrition, Mahidol University, Thailand. The study protocol complied with the Declaration of Helsinki and the International Conference on Harmonization Guidelines for Good Clinical Practice (ICH-GCP), and was approved by the Ethical Committees for Mahidol University Central Institutional Review Board (MU-CIRB) with protocol No. 2021/289.0106 Approval No. 2021/169.1507. The protocol namely “Novel approach to prevent sarcopenia using a combination of eicosapentaenoic acid (EPA) and branch-chained amino acids (BCAAs): a pilot study in elderly people with inadequate protein intake” was registered in the Thai Clinical Trials Registry (TCTR) with protocol No. TCTR20230116005. It can be accessed at https://www.thaiclinicaltrials.org/#. All participants provided written informed consent before enrollment.

### Participants

This study recruited 60–90-year-old healthy people who had no systemic diseases or had stable and controlled diseases, e.g., diabetes, hypertension, or dyslipidemia. Elderly with controlled diabetes must have HbA1C <9%, FBS <180 mg/dL, and no drug adjustment throughout the period of data collection. Subjects should have normal liver and renal function based on ALT <60 IU/L and eGFR ≥ 60 ml/min. All of the participants were evaluated for the risk of malnutrition leading to sarcopenia. They noticeably had inadequate protein intake; consuming <0.5 g/ kg body weight during the past 7 days or <0.7 g/ kg body weight during the past 14 days. The criteria for inadequate protein intake were set up according to the ESPEN guidelines (12). Inadequate protein intake was defined as consuming <50% of the nutritional goal within 7 days or experiencing lower intake than 70% of the individual target constantly, as assessed via dietary recall at screening. The goal for protein intake was 1 g/ kg body weight per day (12). The participants were excluded if they had communication problems, cancer, liver disease, renal disease at stages 3 to 5, Alzheimer's disease, malabsorption such as short bowel syndrome, alcoholism, inflammatory bowel disease (IBD), and pancreatitis, allergic to fish, soybean, peanut, and milk, blood coagulation problems (e.g., taking anti-coagulants (e.g., warfarin), or having some diseases such as thrombocytopenia or leukemia). The participants were acknowledged of the purpose and protocol of the research and given information documents. Then, the volunteers signed a written consent to participate in this research project.

### Sample size and power

The sample size was calculated by using G power V.3.1.9.4. Power of 0.8, theoretical large effect size (0.4) for one-way ANOVA of four groups, and a level of significance (α) of 0.05, was used for calculation. The non-centrality parameter combination (NPC) is 12.16 with a critical F-value of 2.73 which contributed to the total of 76 subjects (19 in each group). Adding the anticipated 10% drop-out, the total sample size was 84 participants (21 in each group).

### Study design blinding, random allocation, and procedures

The blinded randomized control procedure was achieved. All participants were blinded from the intervention assignment by labeling the package of complete nutrition drinks with A, B, C, and D. The random group assigner, data collector, laboratory analyzer, and statistical analyzer were different persons. As shown in Supplementary Figure S1, the 84 participants were randomly assigned into 4 groups equally with minimization using matched age, sex, body mass index, and amount of protein and energy intakes. The four groups received different formulas of complete nutrition drinks: (1) control formula, (2) fortified with 2.2 g EPA, (3) fortified with 2.2 g EPA and 5 g BCAA (2:1:1 of Leu: Ile: Val), and (4) fortified with 2.2 g EPA plus 5g BCAA (4:1:1 of Leu: Ile: Val). Each participant was asked to consume two sachets of the complete nutrition drinks provided 500 kcal/day and 25 g protein to ensure decent energy and protein for daily requirements. Clinical and laboratory outcome parameters were measured at 0 and 3 weeks after interventions. Each participant was asked to do overnight fasting before 10 ml of blood was collected during each visit. The blood was divided into several portions; plasma for measuring complete blood count (CBC), glucose, cholesterol, liver, and kidney function markers, muscle synthesis/ degradation markers, EPA, BCAA, carnosine histidine, and beta-alanine, and serum for measuring cytokines.

### Interventions

The complete nutrition drinks were produced as described (27). Briefly, the placebo control formula liquid was made from Blendera-MF powder (Thai Otsuka Pharmaceutical Co., Ltd., Samut Sakhon, Thailand). The fortified formulas, fish oil containing EPA (PronovaPure^®^, BASF Pharma, Florham Park, NJ, USA) and/ or BCAAs (leucine, isoleucine, and valine from Ajinomoto Co., Ltd. (Thailand), were added and mixed into the control formula liquid. Each formula liquid was filled in the retort pouch for 250 mL per sachet. The pouches were sterilized by retort at 116°C for 25 min. This condition was selected to achieve a lethality value (F0) and ensure sterility as described (27). The products passed microbial safety for low-acid canned food according to Codex standards. Supplementary Table S1 describes the ingredients of all formulas. Nutrition values are as described (27). Our previous study showed that retort sterilization resulted in a 30% reduction in EPA content with no effects on the amount of BCAA (27). Thus, in this study, we increased the amount of EPA used as a raw material by 30%, resulting in a product that, after sterilization, comprises 1.1 g of EPA and 2.5 g BCAA per 250 mL sachet. Consuming two sachets per day will provide 2.2 g of EPA and 5 g of BCAA as designed.

Consented participants who met inclusion criteria and agreed to join the study were provided with complete nutrition drinks according to the random assignment. If the participants also had inadequate energy intake from regular food, they were instructed to consume the drink as a supplement in addition to regular meals. However, if the participants already had adequate energy intake from regular food, they were asked to consume the drink as a replacement for breakfast. All participants continued to consume two 250 mL sachets of the assigned drinks daily for 3 weeks. Furthermore, all of them were instructed to consistently perform arm muscle exercises by squeezing the given resistance rubber handgrip ring (Decathlon^®^) twice daily for 10 min/ set. All participants were advised to maintain their dietary behavior throughout the study.

### Monitoring

To monitor dietary behavior, all participants were asked to record in their subject diaries throughout the study. They filled up dietary records of their intakes every week. In each week of recording, 2 weekdays and 1 weekend data were obtained. To monitor safety, all participants were asked to record any adverse symptoms associated with the supplementation in subject diaries. In addition, routine blood chemistry including complete blood count (CBC), glucose, lipid profile, and liver and kidney markers were measured at 0 and baseline and 0 and 3 weeks after interventions. On a follow-up visit, all subjects were also interviewed for adverse events.

### Compliance

To ensure compliance in consuming the assigned formula, participants were asked to make daily records of consumption and return the empty sachets at the follow-up visits. Furthermore, plasma levels of EPA and BCAA were measured to ensure compliance (see next section for the laboratory methods).

### Outcome measurement

#### Body composition and muscle strength

Body weight, muscle, and fat mass values were determined by bioelectrical impedance analysis (BIA) using the Tanita BC-545 body composition analyzer (Tanita Cooperation, Tokyo, Japan). Handgrip muscle strength of the right and left arms was determined by a hand-held dynamometer (28) using Jamar Plus + Digital 563213 (Lafayette Instrument Company, IN, USA).

### Laboratory analyses

#### Plasma preparation for chemical evaluation via LC-MS/MS

The blood samples were collected. Blood samples were centrifuged at 1000 rpm at room temperature for 10 min. The upper layer of plasma was collected and stored at −20^o^C until analyses.

### Analysis of EPA via LC-MS/MS

Plasma EPA levels were determined by using a method described previously (29). An aliquot of 100 μL of plasma was used to extract lipid with hexane/isopropanol. The extraction began with 3:2 volume by volume of hexane: isopropanol at 1:10 volume by volume of plasma to solvent. After mixing well with the vortex, the mixture was incubated at −20°C for 10 min, followed by centrifugation at 14,000 g at 4°C for 5 min. Then, the hexane extract layer was collected and dried to remove solvents using a SpeedVac concentrator (CentriVap Benchtop Vacuum Concentrators, Labconco, USA) at room temperature for 15 min. One milliliter of 80% methanol was added to the extracted plasma. Nine hundred microliters of the extract were alkaline hydrolyzed by adding 100 μL of 0.3 M KOH in 80% MeOH and incubating at 80°C for 30 min. After the sample was cooled down, 10 μL of formic acid was added to adjust the pH. One milliliter of hexane was added for the extraction of fatty acids and mixed for 5 min, followed by centrifugation at 1000 g. The hexane layer was collected and evaporated using a SpeedVac concentrator to remove the solvent. Then, the sample was reconstituted with 1 mL of 80% methanol and filtered through a 0.2 μm Nylon filter prior to liquid chromatography-tandem mass spectrometry (LC-MS/MS) analysis. For calibration curve generation, 0, 125, 250, 500, 750, and 1000 ng/mL of EPA in 80% methanol were used. An internal standard, EPA-d5 (MaxSpec Cayman) was added to all plasma samples and standard solutions equally at the beginning of sample preparation.

Liquid chromatography was completed with Ultimate 3000 Ultra High-performance liquid chromatography (UHPLC, Thermo Scientific, Waltham, MA, USA) well-assembled with a Hypersil GOLD™ C18 column (100 × 2.1 mm, particle size 1.9 μm) at 50°C. The prepared mobile phases were acetonitrile (mobile phase A) and 5 mM ammonium acetate in water (mobile phase B), respectively. The flow rate condition was established at 0.6 mL/min with 10 min period per injection. The mobile phase was regulated in a gradient form, with the percentage of mobile phase A being 0–6.5 min: 60% A, at 6.5–9 min: 98% A, and at 9–10 min: 60% A. Each single injection quantity was 5 μL. The retention times at 1.7 min were similar to EPA and EPA-d3. Mass spectrometry was connected to liquid chromatography with TSQ Quantis triple quadrupole mass spectrometer (Thermo Scientific, USA). The mass spectrometry setting was negative ion electrospray ionization (-ESI) with spraying voltage at 3500 V under the N2 sheath. Sheath and auxiliary gases were 30 and 15 arbitrary (Arb) units, respectively. The temperature of the ion transfer tube (ITT) was 325°C, while the vaporizing temperature was higher at 350°C. The selected reaction monitoring (SRM) mode for real-time analysis of multiple masses mass was executed for spectrometer analysis. The mass-to-charge ratio (*m/z*) of EPA precursor was 301. The quantified product mass of EPA was *m/z* 257. A collision energy of 12 V was used for the transition. The confirmed product mass for EPA was *m/z* 203.3 with a collision energy of 13 V for the transition. The mass-to-charge ratio (*m/z*) of EPA-d5 precursor and quantified product masses was 306.3. The confirmed product mass for EPA-d5 was *m/z* 262.3 with a collision energy of 12 V for the transition.

### Analysis of plasma BCAA via LC-MS/MS

Plasma BCAA levels were determined by using a method described previously (30). An aliquot of 100 μL of plasma was mixed with 10 μL of 30% sulfosalicylic acid in an Eppendorf tube. The mixture was mixed well and kept under 4°C for 30 min (30). The mixture was separated via centrifugation at 12000 rpm for 5 min. A measure of 50 μL of supernatant was collected. The sample was filtered through a 0.2 μm Nylon filter and kept in a glass vial for further analysis. Standards of valine, isoleucine, and leucine used in this study are the metabolomics amino acid mixtures unlabeled standard (MSK-A2-US-1.2: Cambridge Isotope Laboratories, Inc., USA). Internal standards including 13C/ 15N-valine, 13C/15N-isoleucine, and 13C/15N-leucine were supplied by stable isotope-labeled metabolomics amino acid mixtures (MSK-A2-1.2: Cambridge Isotope Laboratories, Inc., USA).

Liquid chromatography was accomplished with Ultimate 3000 Ultra High-performance liquid chromatography (UHPLC, Thermo Scientific, Waltham, MA, USA) well-assembled with a Hypersil GOLD™ C18 column (100 × 2.1 mm, particle size 1.9 μm) at 30°C. The mobile phase solution for chromatography was prepared with 95% of methanol: 5% of 20 mM Ammonium formate in water. The analytical solution had a flow rate of 0.3 mL/min and a run time of 5 min per injection. The individual volume was 5 μL per single injection. The retention times according to the protocol of mixed amino acids standard of valine, ^13^C/ ^15^N-valine, isoleucine, ^13^C/ ^15^N-isoleucine, leucine, and ^13^C/ ^15^N-leucine were 1.21, 1.19, 1.81, 1.85, 1.98, and 2.01 min, correspondingly. Mass spectrometry (MS) was connected to liquid chromatography with TSQ Quantis triple quadrupole mass spectrometer (Thermo Scientific, USA). The mass spectrometry condition was set to positive ion electrospray ionization (+ESI). The spraying voltage was stable at 3500 V under N2 Sheath. The controlled sheath, auxiliary, and sweep gases were 50, 10, and 1 Arbs. The precise temperatures of 325°C and 350°C condition were marked for ion transfer tube (ITT) and vaporizing conditions. The selected reaction monitoring (SRM) condition was the specific mode for mass analysis in the concurrent investigation of all masses. The mass-to-charge ratio (*m/z*) of valine precursor and quantified product masses were 118 and 72, separately. The transition was carried at an exact collision energy of 10 V. Masses were confirmed with defragmented mass product mass for valine was *m/z* 55 with a collision energy of 21 V for the transition. The mass-to-charge ratio (*m/z*) of ^13^C/ ^15^N-valine precursor and exact quantified defragmented mass was 306.3 and 262.3, correspondingly. The collision energy applied for the transition was 11 V for valine. The mass-to-charge ratio (*m/z*) of isoleucine precursor and quantified product masses was 132 and 86, correspondingly. A collision energy of 10 V was used for the transition. The confirmation product mass for isoleucine was m/z 69 with a collision energy of 17 V for the transition. The mass-to-charge ratio (*m/z*) of ^13^C/ ^15^N-isoleucine precursor and quantified product form was 139.2 and 74, individually. The collision energy of 18 V was used for the transition. The mass-to-charge ratio (*m/z*) of leucine precursor and quantified product atomic mass was 132 and 86, separately. The collision energy applied for the transition was 10V for leucine. The confirmation product mass for isoleucine was *m/z* 44 with the collision energy of the transition. The collision energy applied for the transition was 22 V for isoleucine. The mass-to-charge ratio (*m/z*) of ^13^C/ ^15^N-leucine precursor and quantified product form was 139.2 and 92, correspondingly. The collision energy of applied for the transition was 18 V for valine. The mass-to-charge ratio (*m/z*) of 13C/15N-valine precursor and quantified product form was 139.2 and 92, distinctly.

### Analysis of plasma carnosine, β-alanine, and histidine via LC-MS/MS

Plasma carnosine, β-alanine, and histidine levels were determined by using a method described previously (31). Before using plasma samples, they were fully thawed by leaving them at room temperature. Sample preparation begins by combining 400 μL of methanol with 100 μL of plasma. Then, the mixed solution was centrifuged at 13,000 rpm for 15 min. The concentrated sample was evaporated with a SpeedVac concentrator to remove the solvent. The mobile phase A was prepared from 0.1% formic acid, which was applied to adjust the volume of the sample to a final capacity of 50 μL as reconstitution. The final solution was filtered through a 0.2 μm Nylon filter. Samples were kept in a glass ampoule for chemical evaluation. The liquid chromatography was processed using Ultimate 3000 Ultra High-performance liquid chromatography (UHPLC, Thermo Scientific, Waltham, MA, USA) well-assembled with a Hypersil GOLD™ C18 column (100 × 2.1 mm, particle size 1.9 μm) at 30°C. Mobile phase solution for chromatography was prepared with MS-grade water with 0.1% formic acid (mobile phase A) and acetonitrile with 0.1% formic acid (mobile phase B). The analytical solution was controlled at a flow rate of 0.2 mL/min and a run time of 5 min per injection. The individual volume was 5 μL per single injection. The retention time was noticed. The adjusted gradient of mobile phases A and B by time laps is presented in Supplementary Table S2.

Mass spectrometry was connected to liquid chromatography with TSQ Quantis triple quadrupole mass spectrometer (Thermo Scientific, USA). The MS condition was set to positive ion electrospray ionization (+ESI) to identify mass spectra and transitions of carnosine, β-alanine, and histidine. The spraying voltage was stable at 4600V under N2 Sheath. The controlled sheath and auxiliary gases were 50 and 10 Arbs. The precise temperatures of 300°C and 350°C condition were marked for ion transfer tube (ITT) and vaporizing conditions. For the concurrent investigation of all masses, the selected reaction monitoring (SRM) condition was the specific mode for mass analysis. The mass-to-charge ratio (*m/z*) of β-alanine precursor and quantified product masses was 90.31 and 29.964, separately. The transition was carried at an exact collision energy of 13.76V. The mass-to-charge ratio (*m/z*) of histidine precursor and quantified product masses was 156.062 and 82.833, separately. The transition was carried at an exact collision energy of 24.29V. The confirmed fragment mass of 110 was further analyzed by applying a collision energy of 13.72. The mass-to-charge ratio (*m/z*) of carnosine precursor and quantified product masses was 227.2 and 110.054, separately. The transition was carried at an exact collision energy of 22.355 V. The confirmed fragment mass of 156.125 was further analyzed by applying a collision energy of 15.236.

### Analysis of IL-6 and IL-10

#### Serum preparation for interleukin evaluation via ELISA

The blood samples were obtained in a serum vacutainer tube containing a clot activator, after overnight fasting. After the blood was clotted, each sample was centrifuged at controlled room air condition of 1,000 rpm for 10 min. The lower layer of solution was collected after centrifugation. Serum was processed and stored at −20^o^C until evaluation. The prepared serum was thawed by leaving it at room temperature before being used.

### ELISA procedure for the analysis of IL-6 and IL-10

The concentrations of human IL-6 and IL-10 within serum sample were evaluated by using the method of enzyme-linked immunosorbent assay (ELISA) kits (Catalog No. ab46042 and ab46034, correspondingly; Abcam Co., Cambridge, MA, USA), as described previously (32). The assays were performed according to the kits' instructions. For the test, 100 μL each of the given standard or prepared plasma samples was added to each well of the 96-well plate coated with the antibody of IL-6 or anti–IL-10. Then, 50 μL of biotinylated anti–IL-6 or anti–IL-10 antibody, which accounted for the secondary antibody, was added to the well, and the mixture was incubated for 2 h at room temperature. After that, the samples were washed three times with 300 μL of wash buffer. Next step, 100 μL of Streptavidin-HRP solution was added into the previous mixture. The solution was incubated for 30 min. After three washes with buffer, 100 μL of chromogen TMB substrate was added, and the samples were incubated in the dark for 15 min. Finally, 100 μL of stop reagent was added to end the fraction. The concentrations were determined by a microplate reader capable of measuring absorbance by setting the wavelength at 450 nm (Epoch, BioTek Industries, Highland Park, USA). Calibration curves of IL-6 ranging from 0 to 50 mg/L were used for the quantitation of the IL-6 level in serum. Calibration curves of IL-10 ranging from 0 to 400 mg/L were used for the quantitation of IL-10 level in serum.

### Statistical analysis

A per-protocol analysis was performed. Graphing and the following statistical analyses were conducted using Graph Pad Prism Software V. 7. Demographic characters between study and control groups were analyzed using the chi-square test. All parameters were compared between baseline and after intervention in the same group by using paired *t-*test or Wilcoxon signed rank test depending on the normality of the data. Comparison of changes over time of each parameter between study and control groups was performed using two-way repeated measure ANOVA followed by Tukey's multiple comparison tests.

## Results

### Participants' flow diagram

**Figure 1:**
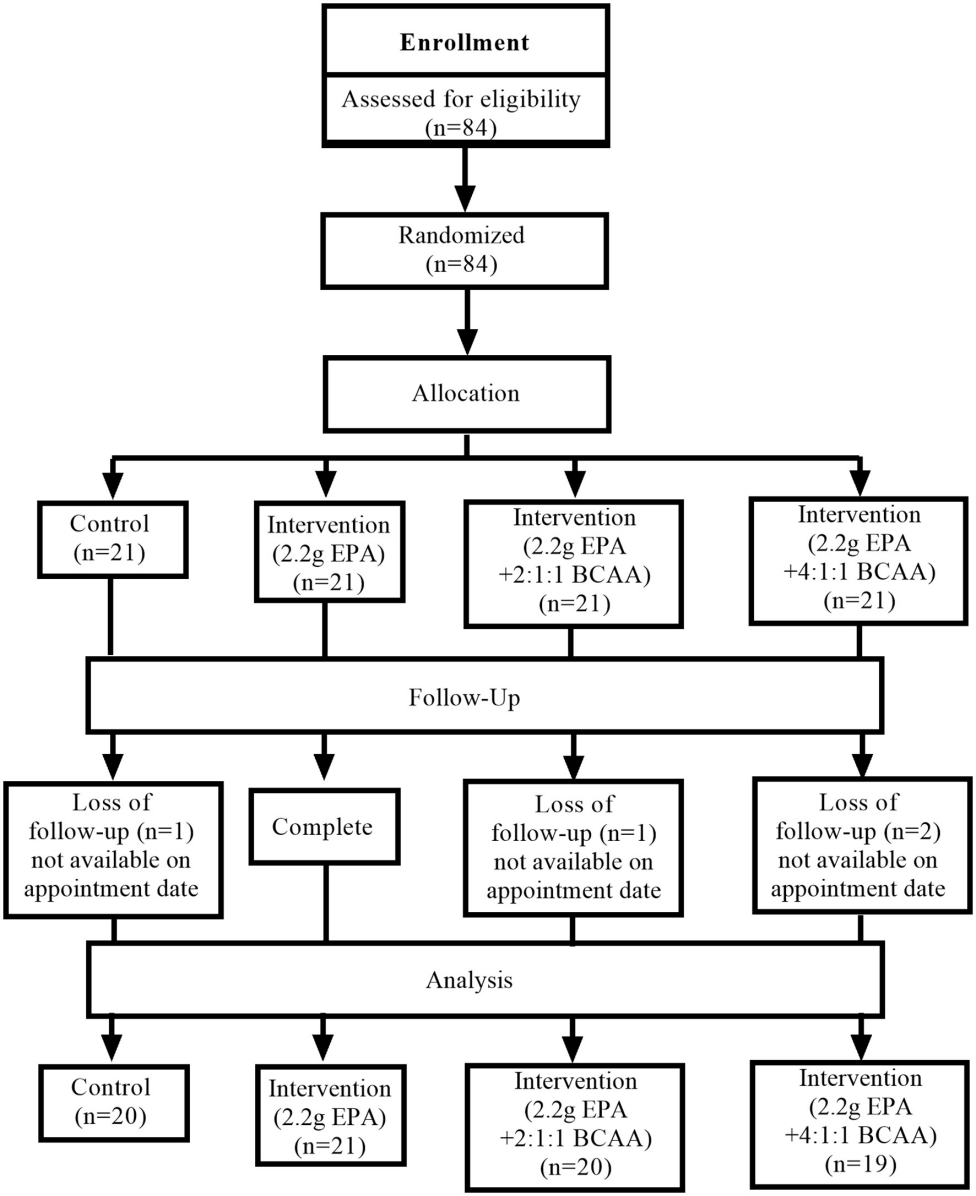
Conceptual framework.

This study was conducted from August 2020 to July 2021. Figure 1 shows the Consolidated Standards of Reporting Trials (CONSORT) participants' flow diagram. Eighty-four volunteers were recruited for the study and all of them passed the screening. All 84 participants were randomly assigned into four groups (n = 21, for each group) receiving different medical formulas; (1) control complete nutrition drink, (2) fortified with only 2.2g/day of EPA, (3) fortified with 2.2g/day of EPA and 5g/day of BCAA 2:1:1 of Leu: Ile: Val, and (4) fortified with 2.2g/day of EPA and 5g/day of BCAA 4:1:1 of Leu: Ile: Val. Few participants in certain groups lost follow-up. The final data were collected from 20, 21, 20, and 19 participants in groups 1, 2, 3, and 4, respectively.

### Participants' characteristics

The baseline characteristics of participants in each group are shown in Table 1. The mean ages of participants were 67.86 ± 4.62, 65.15 ± 4.09, 68.05 ± 5.11, and 66.60 ± 5.28 years old for groups (1), (2), (3), and (4), respectively. There was no significant difference in age among groups (*p* = 0.239). Most of the participants were in a greater proportion of females than males. There was no significant difference in gender distribution among groups (*p* = 0.931). The mean initial body mass indices were not different among groups (*p* = 0.929). Furthermore, there were no significant differences among groups for systemic diseases, medications, muscle mass, % fat, handgrip strength, and average energy and protein intakes.

**Table 1:** Baseline characteristics of participants.

**Characteristic**	**Number (%)**				***p*-value**
	**Group 1 control (*****n** =* **20)**	**Group 2 EPA(*****n** =* **21)**	**Group 3 EPA**+**BCAA (2:1:1) (*****n** =* **20)**	**Group 4 EPA**+**BCAA (4:1:1) (*****n** =* **19)**	
Age	67.86 ± 4.62	65.15 ± 4.09	68.05 ± 5.11	66.60 ± 5.28	0.24
Sex Male Female	4 (19.05%) 17 (80.95%)	4 (20%) 16 (80%)	4 (21.06%) 15 (78.94%)	4 (20%) 16 (80%)	0.93
Systemic diseases None DM/ DM+DLP DLP	11 (52) 4 (19) 6 (29)	11 (55) 3 (15) 6 (30)	8 (42) 3 (16) 8 (42)	8 (40) 5 (25) 7 (35)	0.13
Medication use None Yes^a^	11 (52) 10 (42)	11 (55) 9 (45)	8 (42) 11 (58)	8 (40) 12 (60)	0.88
BMI	22.9 ± 4.1	23.1 ± 4.6	22.2 ± 4.8	22.1 ± 3.9	0.93
Muscle mass (kg)	37.8 ± 8.1	37.3 ± 5.9	35.9 ± 5.7	37.8 ± 6.9	0.78
% Fat	28.6 ± 7.4	30.3 ± 9.8	27.8 ± 9.1	26.1 ± 9.6	0.50
Handgrip strength (Right arm)	22.2 ± 5.8	24.0 ± 5.5	22.8 ± 5.9	25.0 ± 5.3	0.28
Handgrip strength (Left arm)	21.2 ± 6.5	21.9 ± 6.6	21.7 ± 4.3	24.1 ± 5.7	0.40
Average energy intake	1226 ± 185.6	1300 ± 318.9	1192 ± 193.5	1201 ± 469.5	0.68
Average protein intake	52.97 ± 12.13	55.41 ± 12.40	52.09 ± 14.09	57.89 ± 19.60	0.61

Data were expressed as mean ± standard deviation (SD). The statistical differences among groups were analyzed using one-way ANOVA. ^a^Blood sugar control/ blood lipid control drugs.

### Changes in body weight, BMI, muscle mass, fat mass, and visceral fat

**Figure 2:**
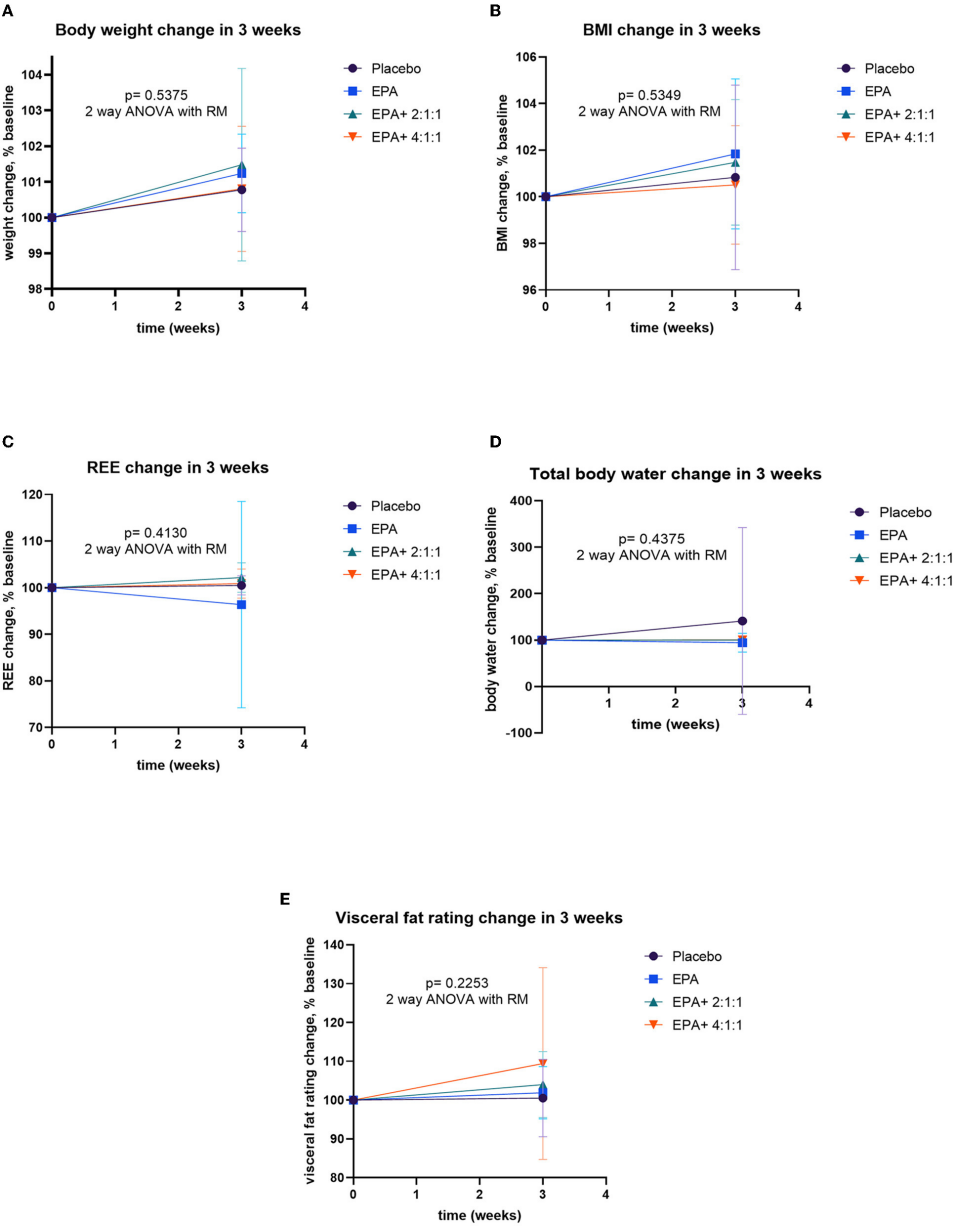
Percent changes of body composition before and after receiving specified interventions for 3 weeks. Line plots show mean and standard deviation (SD) of body weight **(A)**, BMI **(B)**, resting energy expenditure **(C)**, water **(D)**, and visceral fat **(E)**. The differences among groups were analyzed using two-way ANOVA with repeated measure.

**Figure 3:**
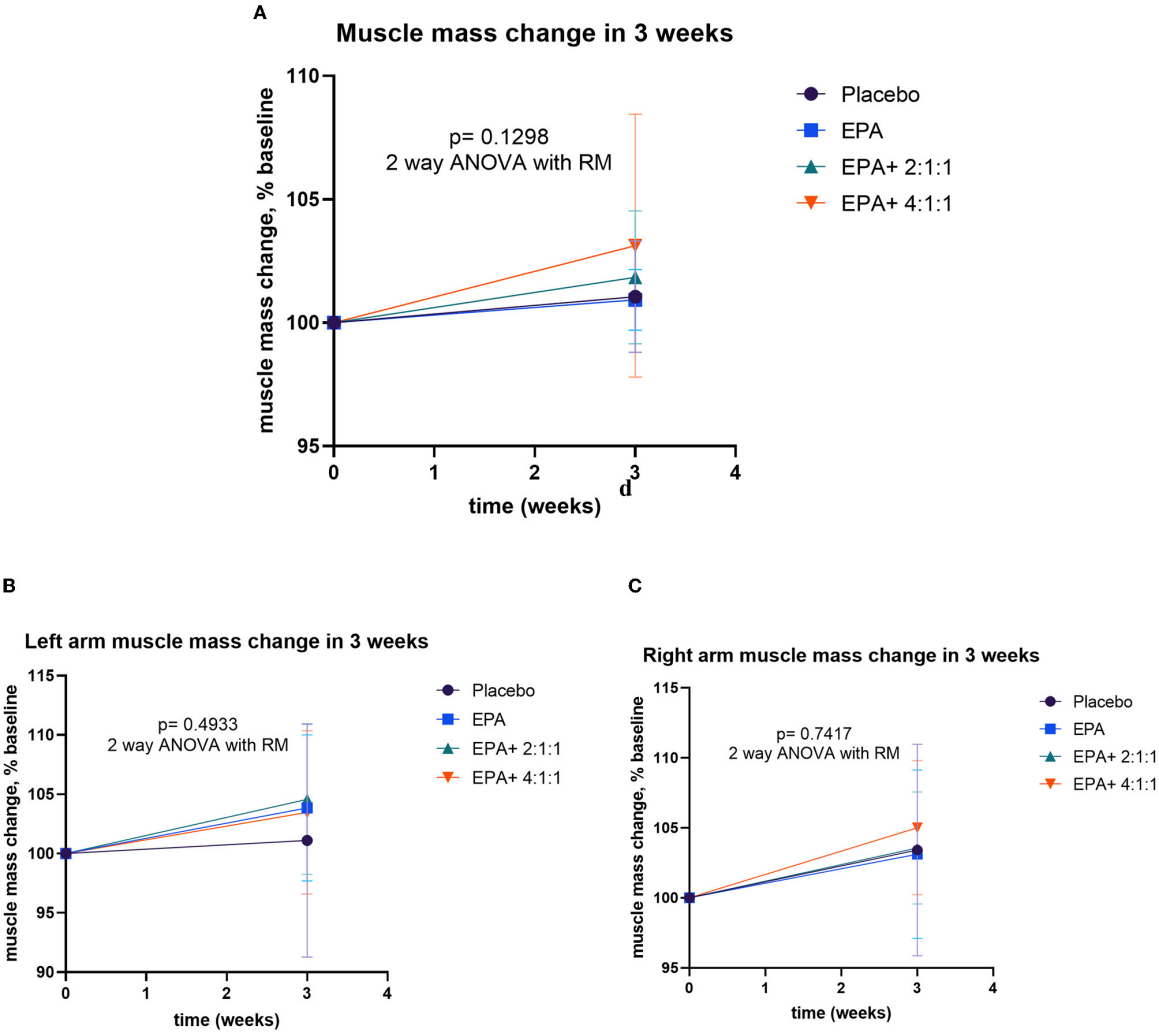
Percentage changes in muscle mass, left arm muscle mass, and right arm muscle mass before and after receiving specified interventions for 3 weeks. Line plots show the mean and standard deviation (SD) of total muscle mass **(A)**, left arm muscle mass **(B)**, and right arm muscle mass **(C)**. The differences among groups were analyzed using two-way repeated measure ANOVA.

**Figure 4:**
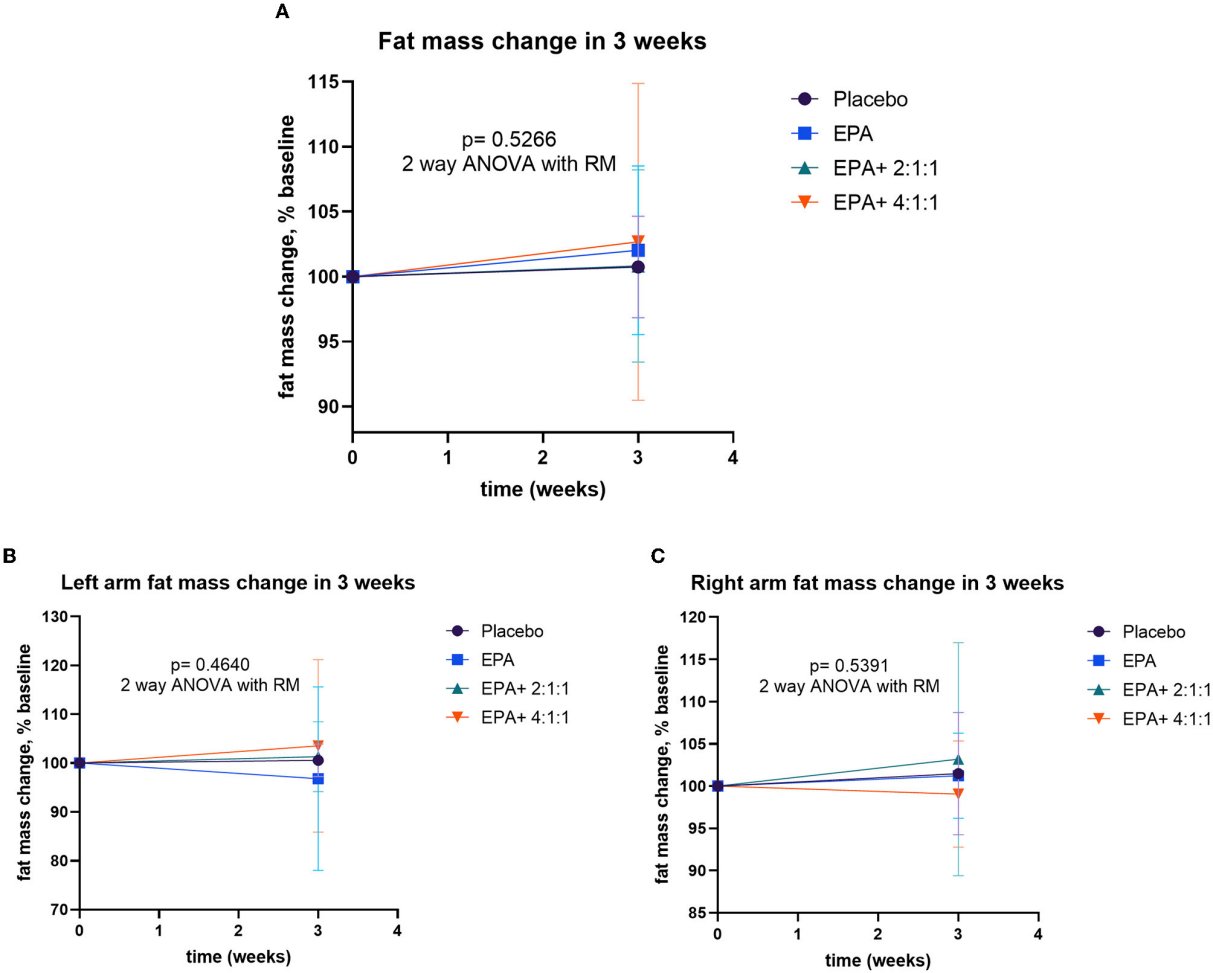
Percentage changes in fat mass, left arm fat mass, and right arm fat mass before and after receiving specified interventions for 3 weeks. Line plots show mean and standard deviation (SD) of total fat mass **(A)**, left arm muscle mass **(B)**, and right arm muscle mass **(C)**. The differences among groups were analyzed using two-way repeated measure ANOVA.

After participants drank the given complete nutrition drinks of each group along with performing the hand grip exercise, all of them were measured for the changes in body composition via BIA. The changes in body weight, BMI, muscle mass, fat mass, and visceral fat before and after receiving complete nutrition drinks from each group of participants are shown in Table 2. The body weight of groups 2 and 3 and the BMI of group 2 increased significantly after consuming the assigned interventions (*p* < 0.01 and *p* < 0.05, respectively). Interestingly, as shown in Table 2, the average right arm muscle mass was slightly increased in all groups. The statistically significant improvement was only observed in groups 3 and 4, which received complete nutrition drink fortified with 2.2 g/day of EPA and 5 g/day of BCAA 2:1:1 and 4:1:1 of Leu: Ile: Val, respectively (*p* < 0.05 and *p* < 0.01, respectively). No significant differences in total muscle mass, left arm muscle mass, % fat, and visceral fat rating were found between before and after interventions. When comparing changes (as % baseline) after interventions among groups, there were no statistically significant differences in body weight, BMI, resting energy expenditure (REE), total body water, visceral fat rating, total, left and right arm muscle mass, total, and left and right arm fat mass among all groups (Figures 2–4). Interestingly, Figure 3 shows a pronounced higher increase in the total muscle mass and the right arm muscle mass after receiving the fortified formula with EPA and 5g/day of BCAA 4:1:1 of Leu: Ile: Val. However, two-way ANOVA showed no statistically significant difference from other groups.

**Table 2:** Comparison of body weight, BMI, muscle mass, fat mass, and visceral fat before (visit 1) and after (visit 2) receiving the assigned drinks and handgrip practice for 3 weeks.

**BodyComposition**	**Group 1 control** (***n =***** 20)**			**Group 2EPA** (***n =*** **21)**			**Group 3 EPA +BCAA (2:1:1)** ( ***n =*** **20)**			**Group 4 EPA +BCAA (4:1:1)** (***n =*** **19)**		
	**Visit 1**	**Visit 2**	***p*** **-value**	**Visit 1**	**Visit 2**	***p*** **-value**	**Visit 1**	**Visit 2**	***p*** **-value**	**Visit 1**	**Visit 2**	***p*** **-value**
Body weight	56.6 ± 13	57.1 ± 13.1	0.10	57.5 ± 11.4	58.2 ± 11.4	^**^	56.4 ± 14.5	57.1 ± 14.3	^**^	55.6 ± 11.9	56.1 ± 12.0	0.11
BMI (kg/m^2^)	23.0 ± 4.2	23.1 ± 4.3	0.58	23.1 ± 4.6	23.5 ± 4.5	^*^	22.4 ± 4.8	22.7 ± 4.7	0.19	22.2 ± 3.7	22.3 ± 3.8	0.93
Muscle mass (kg)	37.7 ± 8.3	36.4 ± 9.1	0.64	37.3 ± 5.9	37.6 ± 5.7	0.99	36.1 ± 5.8	38.3 ± 7.7	0.21	38.0 ± 7.2	39.1 ± 7.3	0.82
Right arm muscle mass (kg)	1.85 ± 0.55	1.9 ± 0.57	0.91	1.84 ± 0.43	1.9 ± 0.47	0.63	1.86 ± 0.52	1.93 ± 0.55	^*^	1.85 ± 0.53	1.93 ± 0.54	^**^
Left arm muscle mass (kg)	1.76 ± 0.53	1.77 ± 0.54	0.99	1.71 ± 0.41	1.77 ± 0.42	0.14	1.73 ± 0.51	1.8 ± 0.51	0.08	1.73 ± 0.49	1.79 ± 0.51	0.2
% Fat	28.62 ± 7.37	27.76 ± 9.76	0.64	30.3 ± 9.8	30.63 ± 9.5	0.98	27.83 ± 9.28	27.66± 9.31	0.99	25.98 ± 9.39	26.28 ± 9.09	0.99
Visceral fat rating	8.25 ± 4.78	8.1 ± 4.72	0.94	7.9 ± 3.3	8.04 ± 3.35	0.95	7.7 ± 4.54	7.9 ± 4.42	0.86	7.95 ± 5.01	8.42 ± 4.98	0.19

Data were expressed as mean ± standard deviation (SD). The statistical differences between visit 1 (baseline) and visit 2 (3 weeks after intervention) for each group were analyzed using two-way repeated measure ANOVA followed by Sidak's multiple comparison tests (comparing different time points in the same group). The symbols ^*^ and ^**^ mean *p* < 0.05 and 0.01, respectively.

### Changes in handgrip strength

**Figure 5:**
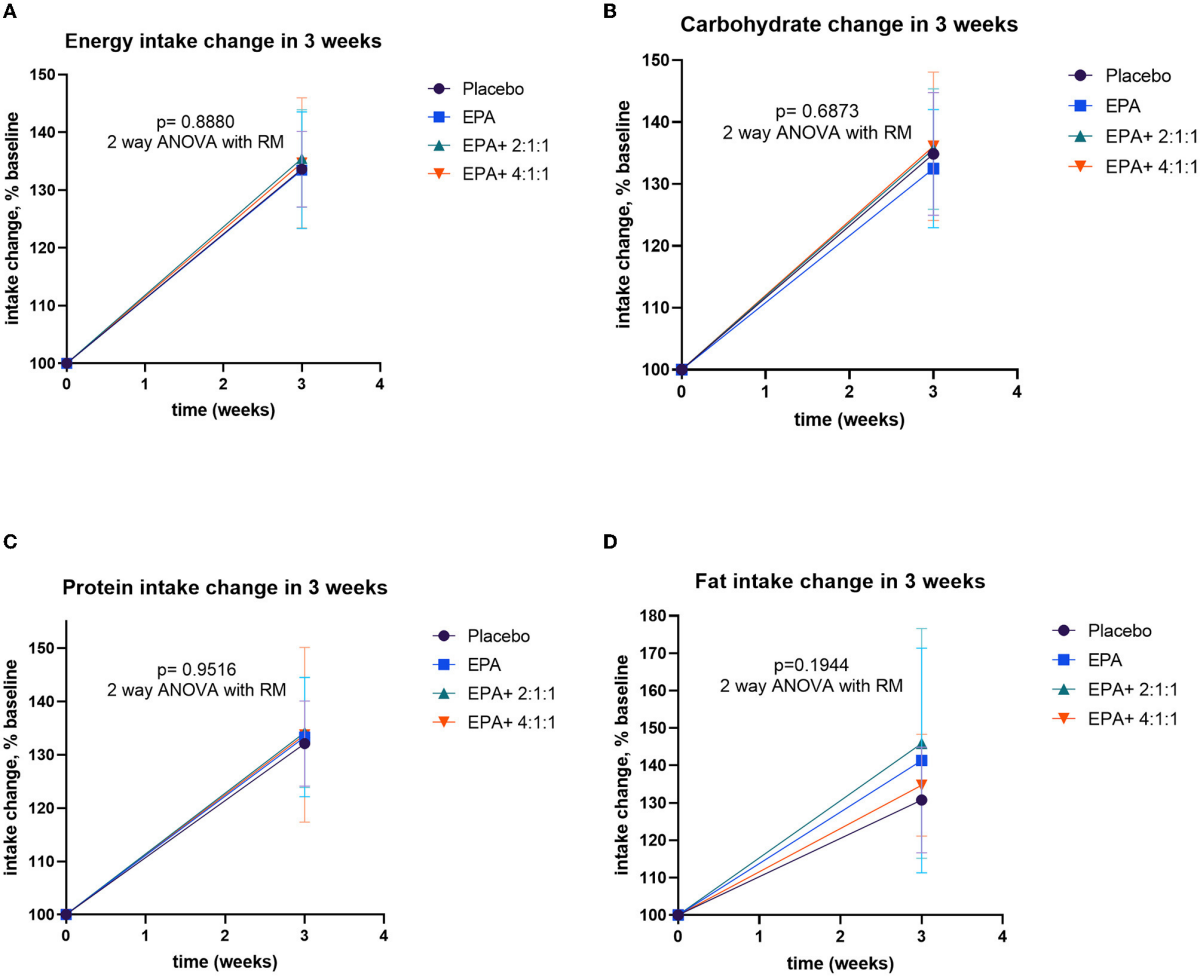
Changes in intakes including energy, carbohydrate, protein, and fat before and after receiving specified interventions for 3 weeks. Line plots show mean and standard deviation (SD) of changes (% of baseline values) in energy **(A)**, carbohydrate **(B)**, protein **(C)**, and fat **(D)** intakes. The statistical differences among groups were analyzed by using two-way repeated measure ANOVA.

During the trial, muscle training with a resistance rubber handgrip was assigned for all participants to perform daily. Moderate training on hands and arm muscles was assigned since it has a low risk of falls in the elderly. To avoid inconsistency, all individuals were asked to continue their routine exercise without extreme exercise throughout the study. A comparison of handgrip strength changes within each group is shown in Table 3. Similar to the result of the right arm muscle mass, a significant increase in handgrip strength of the right arm was found in groups 3 and 4 after consuming the complete nutrition drink fortified with 2.2 g/day of EPA and 5 g/day of BCAA 2:1:1 and 4:1:1 of Leu: Ile: Val, respectively (*p* < 0.05 and *p* < 0.01, respectively). Interestingly, Figure 5 shows a higher increase in the right handgrip strength after receiving the fortified formula with EPA and 5g/day of BCAA 4:1:1 of Leu: Ile: Val. However, two-way ANOVA showed no statistically significant difference from other groups.

**Table 3:** Comparison of handgrip strength within each group.

Handgrip strength^*^	**Group 1 control**			**Group 2 EPA**			**Group 3 EPA**+**BCAA (2:1:1)**			**Group 4 EPA**+**BCAA (4:1:1)**		
	**Visit 1**	**Visit 2**	***p*** **-value**	**Visit 1**	**Visit 2**	***p*** **-value**	**Visit 1**	**Visit 2**	***p*** **-value**	**Visit 1**	**Visit 2**	***p*** **-value**
Right arm	22.19 ± 5.84	23.33 ± 5.45	0.051	23.98 ± 5.5	24.88 ± 5.84	0.14	22.77 ± 5.97	23.92 ± 5.87	^*^	25.03 ± 5.32	26.57 ± 5.62	^**^
Left arm	21.18 ± 6.60	22.42 ± 6.30	0.12	21.86 ± 6.63	22.07 ± 5.91	0.99	21.78 ± 4.39	22.80 ± 5.02	0.27	24.44 ± 5.88	25.35 ± 5.23	0.40

Data were expressed as mean ± standard deviation (SD). The statistical differences between visit 1 (baseline) and visit 2 (3 weeks after intervention) for each group were analyzed using paired t-tests. Each parameter was the average parameter of three times repeated grip measurements. The symbols ^*^ and ^**^ mean *p* < 0.05 and 0.01, respectively.

### Changes in nutrition intake

**Figure 6:**
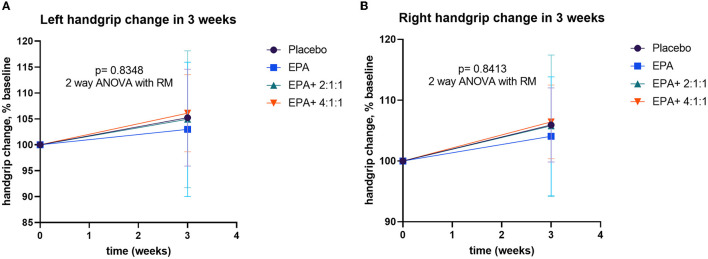
Percentage changes in handgrip strength before and after receiving specified interventions for 3 weeks. Line plots show the mean and standard deviation (SD) of change (% baseline) of the left **(A)** and right **(B)** handgrip strength. The differences within group were analyzed using two-way repeated measure ANOVA.

All of the volunteers were asked to record the daily intake of regular food with assigned complete nutrition drinks. The calculated compliance to the given nutrition formula of groups 1–4 was 81.90, 84.05, 79.76, and 79.88%, respectively, which indicated a non-significantly difference (p=0.647). The nutritional values were calculated from the 3-day record per week to evaluate the differences in intakes before and after the trial. The energy intake and the intakes of macronutrients are significantly greater than those of baseline values in all groups (p=0.015, 0.012, 0.029, and 0.033 for groups 1–4, respectively). The average intakes are expressed in Supplementary Table S3. The comparison of dietary changes among groups was analyzed by using repeated measure ANOVA. The results showed non-significant differences among groups (Figure 6).

### Changes in blood chemistry

The routine blood chemistry laboratory analysis was performed to monitor safety. As shown in Supplementary Table S4, blood biochemical values in all groups were not significantly altered.

### Changes in plasma EPA and BCAA

**Figure 7:**
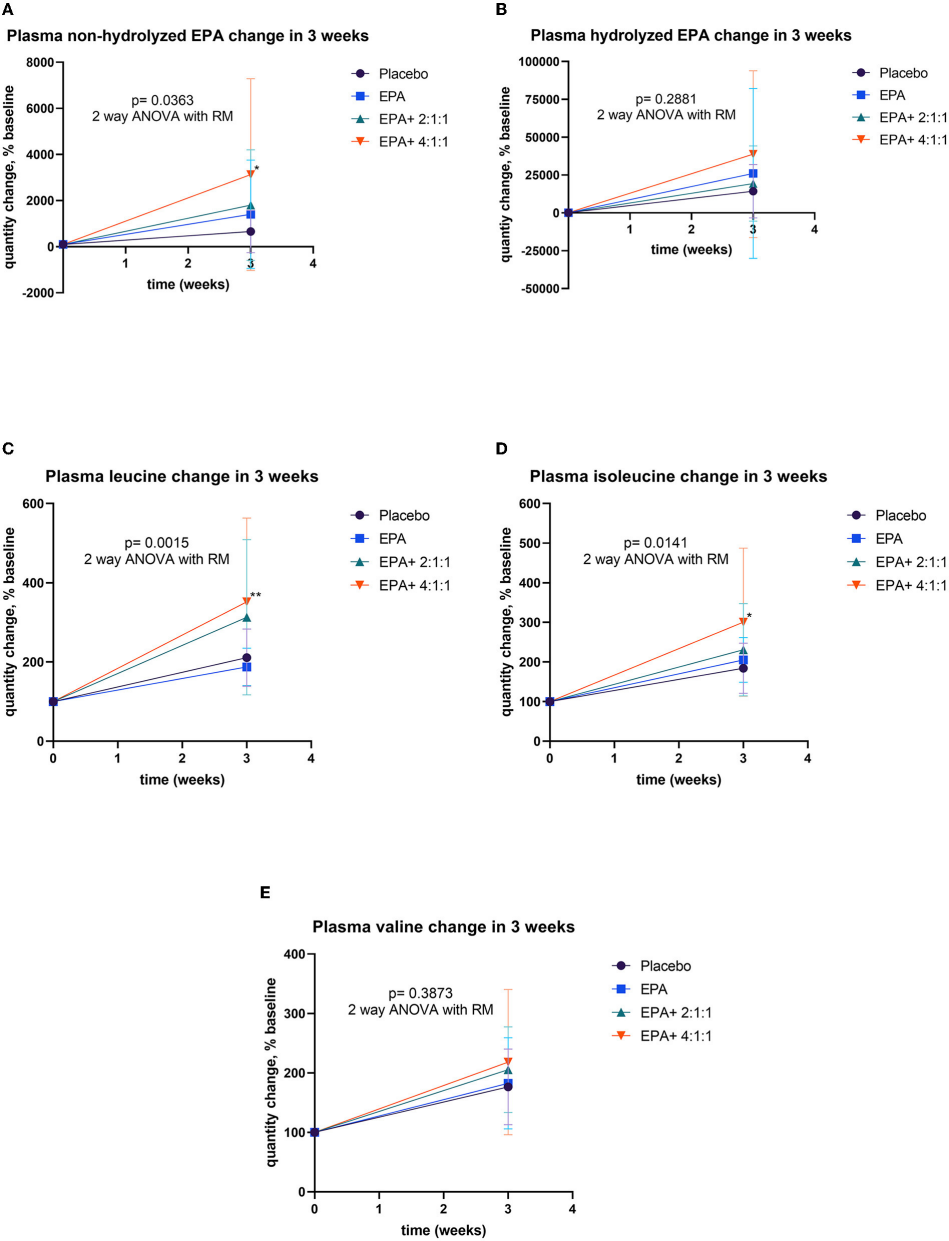
Percentage changes of plasma EPA and BCAA before and after receiving specified interventions for 3 weeks. Line plots show the mean and standard deviation (SD) of change (% baseline) of plasma non-hydrolyzed EPA **(A)**, hydrolyzed EPA **(B)**, leucine **(C)**, isoleucine **(D)**, and valine **(E)**. The statistical differences among groups were analyzed using two-way ANOVA with repeated measure.

The blood samples were drawn at the baseline and after continuous consumption of the assigned complete nutrition drinks for 3 weeks. Plasma was prepared for the analysis of EPA and BCAA by using LC-MS/MS. The comparison of plasma EPA and BCAA concentration via LC-MS/MS within each group is expressed in Table 4. For the non-hydrolyzed EPA evaluation, there was significantly elevated EPA in group 2 after receiving fortified formula containing EPA (p=0.0002) and in group 4 after receiving fortified formula containing EPA + 4:1:1 BCAA (*p* < 0.0001). Moreover, the significant elevation of hydrolyzed EPA was evidenced in group 2 (*p* = 0.078) and group 4 (*p* < 0.0001), respectively. The BCAA was significantly increased in all groups, compared to that of the baseline. Figure 7 shows the comparison of EPA and BCAA among groups. A significant elevation in non-hydrolyzed EPA was observed in the group consuming formula with EPA and 4:1:1 BCAA (*p* = 0.036). The same group also had significantly higher levels of plasma leucine and isoleucine than those of other groups (*p* = 0.001 and *p* = 0.014, respectively).

**Table 4:** Comparison of plasma EPA and BCAA concentrations via LC-MS/MS within each group.

**Chemical analyze**	**Group 1 control**			**Group 2 EPA**			**Group 3 EPA**+**BCAA (2:1:1)**			**Group 4 EPA**+**BCAA (4:1:1)**		
	**Visit 1**	**Visit 2**	***p*** **-value**	**Visit 1**	**Visit 2**	***p*** **-value**	**Visit 1**	**Visit 2**	***p*** **-value**	**Visit 1**	**Visit 2**	***p*** **-value**
Non-hydrolyzed EPA	16.90 ± 18.17	78.39 ± 112.0	0.530	15.73 ± 17.02	165.20 ± 184.7	0.0002	6.74 ± 5.63	101.60 ± 126.2	0.093	8.44 ± 6.99	172.70 ± 147.80	<0.0001
Hydrolyzed EPA	9.34 ± 9.60	688.70 ± 839.0	0.275	9.31 ± 7.86	871.60 ± 1173	0.078	8.33 ± 5.94	784.30 ± 729.0	0.182	5.34 ± 2.83	1551 ± 2100	<0.0001
Leu	165.60 ± 50.05	337.60 ± 124.6	<0.0001	154.20 ± 43.78	277.60 ± 72.45	0.002	142.70 ± 75.09	348.20 ± 132.9	<0.0001	129.10 ± 62.59	398.20 ± 151.8	<0.0001
Ile	33.62 ± 11.85	58.62 ± 18.29	0.0007	32.28 ± 8.65	62.77 ± 12.51	<0.0001	35.80 ± 13.77	75.73 ± 34.76	<0.0001	29.04 ± 14.23	69.53 ± 23.33	<0.0001
Val	117.60 ± 23.16	204.30 ± 83.41	0.008	127.30 ± 24.23	225.60 ± 90.82	0.002	111.70 ± 41.95	232.80 ± 112.4	<0.0001	120.90 ± 27.62	250.80 ± 125.0	<0.0001

Data were expressed as mean ± standard deviation (SD). The statistical differences between visit 1 (baseline) and visit 2 (3 weeks after intervention) for each group were analyzed using paired t-tests.

### Changes in plasma carnosine, β-alanine, and histidine

**Figure 8:**
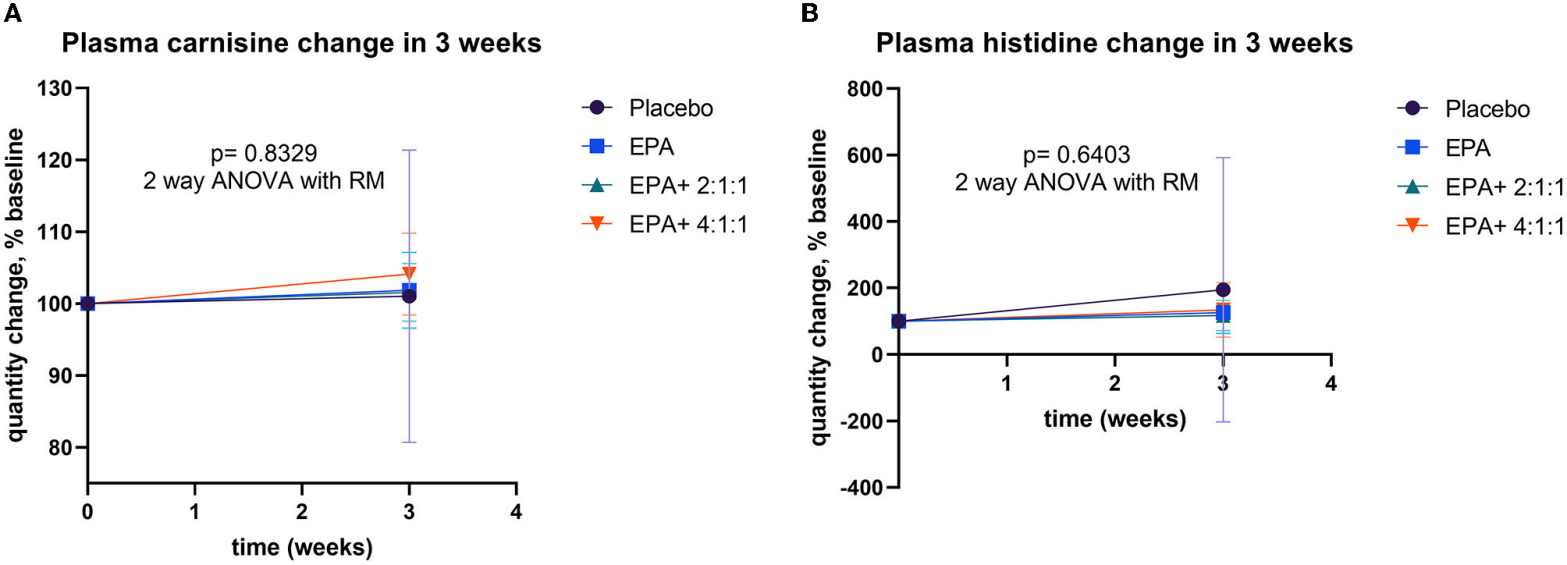
Percentage changes in plasma EPA and BCAA before and after receiving specified interventions for 3 weeks. Line plots show the mean and standard deviation (SD) of change (% baseline) in plasma carnosine **(A)**, and histidine **(B)**. The statistical differences among groups were analyzed using two-way repeated measure ANOVA.

Changes in muscle protein synthesis/ degradation were evaluated by analyzing the amount of plasma carnosine, β-alanine, and histidine. An increase in carnosine indicates a shift toward synthesis, while an increase in β-alanine or histidine represents more muscle protein breakdown. The analysis of plasma carnosine, β-alanine, and histidine concentration via LC-MS/MS within each group is shown in Table 5. Since all data of β-alanine were lower than the lowest quantitation (LOQ) level, the exact amount could not be calculated and reported here. Results of carnosine and histidine showed non-significant changes among groups (Figure 8). Interestingly, Figure 8A shows that group 4 receiving fortified formula with EPA+4:1:1 BCAA has a tendency of increased carnosine level, compared to other groups. Moreover, Figure 8B shows that the plasma histidine levels of the groups receiving fortified formula tended to decrease compared to the placebo control.

**Table 5:** Comparison of plasma carnosine, β-alanine, and histidine concentrations via LC-MS/MS within each group.

**Chemical analyze**	**Group 1 control**			**Group 2 EPA**			**Group 3 EPA**+**BCAA (2:1:1)**			**Group 4 EPA**+**BCAA (4:1:1)**		
	**Visit 1**	**Visit 2**	***p*** **-value**	**Visit 1**	**Visit 2**	***p*** **-value**	**Visit 1**	**Visit 2**	***p*** **-value**	**Visit 1**	**Visit 2**	***p*** **-value**
Carnosine	17.72 ± 4.83	17.29 ± 2.51	0.997	16.50 ± 0.38	16.79 ± 0.73	0.999	16.62 ± 0.44	16.87 ± 0.66	>0.0.999	16.55 ± 0.29	17.23 ± 0.91	0.964
Histidine	199.00 ± 104.00	216.90 ± 117.20	0.999	198.40 ± 104.00	224.00 ± 112.70	0.996	215.90 ± 103.60	239.20 ± 113.40	0.998	210.80 ± 112.70	228.20 ± 124.50	0.999
β-alanine	<LOQ	<LOQ	<LOQ	<LOQ	<LOQ	<LOQ	<LOQ	<LOQ	<LOQ	<LOQ	<LOQ	<LOQ

Data were expressed as mean ± standard deviation (SD). The statistical differences between visit 1 (baseline) and visit 2 (3 weeks after intervention) for each group were analyzed using paired t-tests.

### Changes in serum IL-6 and IL-10

**Figure 9:**
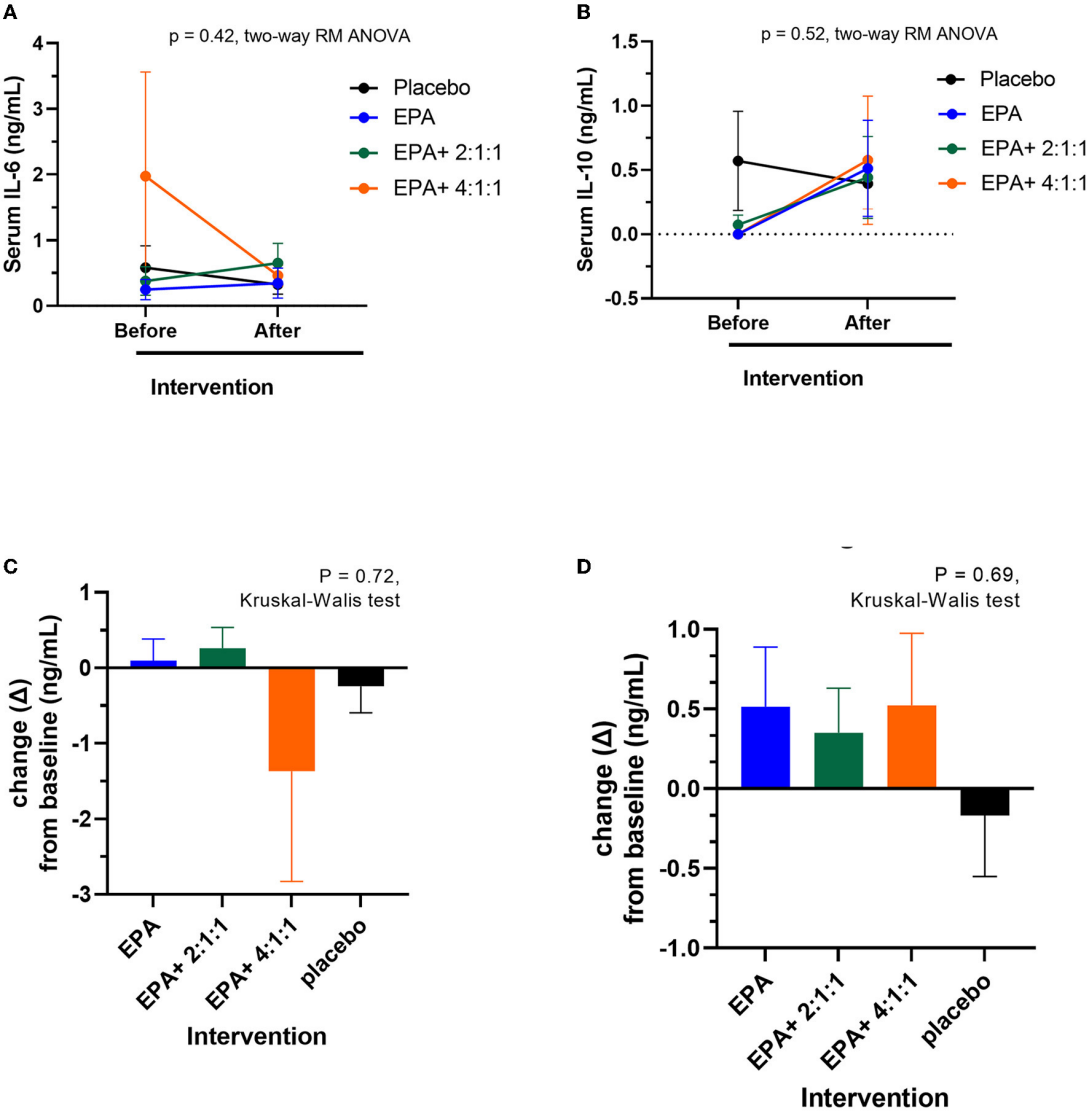
Changes in serum IL-6 and IL-10 levels before and after receiving specified interventions for 3 weeks. Line plots show the mean and standard deviation (SD) of serum IL-6 **(A)** and IL-10 **(B)**, changes (delta = the levels after intervention – the levels at baseline) of IL-6 **(C)** and IL-10 **(D)**. The statistical differences among groups were analyzed using two-way repeated measure ANOVA.

The serum of the participants was separated from a blood sample and examined for inflammation markers via ELISA. The IL-6 represented an inflammation marker, while IL-10 signified an anti-inflammation marker. As shown in Figures 9A, C average IL-6 levels were decreased after receiving fortified formula with EPA+4:1:1 BCAA. However, the difference between the placebo and other groups was not statistically significant. As shown in Figures 9A, C average IL-6 levels were decreased after receiving fortified formula with EPA+4:1:1 BCAA. However, the change was not statistically significantly different from the placebo and other groups. Furthermore, as shown in Figures 9B, D all study groups receiving the drinks fortified with EPA with or without BCAA have a non-significant trend of increased IL-10 plasma levels compared to those of placebo control.

## Discussion

Elevated inflammation and negative nutritional balance are demonstrated to be major causes of developing sarcopenia (13). Nevertheless, it is unknown if energy supplementation plus the combination of anti-inflammatory factors such as eicosapentaenoic acid (EPA) and branched-chain amino acids (BCAAs) would be effective for the prevention of sarcopenia in the elderly with inadequate protein intake. This 3-week randomized control trial revealed that consuming a complete nutrition drink fortified with EPA + BCAA 2:1:1 and 4:1:1 of Leu: Ile: Val for 3 weeks significantly increased the right arm muscle mass and right handgrip strength (*p* < 0.05 and *p* < 0.01, respectively) along with non-significantly elevated carnosine with reduced histidine and increased IL-10 with decreased IL-6 levels. Previous studies showed a beneficial effect of EPA on anti-inflammation and the effect of BCAA supplementation on muscle health. Here we showed that a combination of both could have a trend in improving muscle health and reducing inflammation. This short-term study implies the potential benefits of such a combinatorial approach in preventing sarcopenia in the elderly with inadequate protein intake.

Our finding is consistent with a previous systemic review of 14 randomized controlled trials that show significantly improved muscle mass and muscle strength in elderly people after receiving BCAA-rich nutrition supplementation (33). However, while the systemic review showed a significant effect on total muscle mass, we only see the efficacy on right arm muscle mass and handgrip strength. Though the combination showed better effects than those of the placebo and the EPA only, there were no significant differences between groups. The distinct between our study and those reported in systemic reviews may be due to two reasons; one is that our study population is just at risk of sarcopenia but had not been diagnosed as sarcopenia yet, and the other is the short duration of our study (3 weeks). Previous studies that show statistically significant effects on muscle mass, strength, and performance provided BCAA supplements for at least 5 weeks (34). In the current study of 3 weeks of intervention, a significant increase in muscle mass and handgrip strength of the right arm was observed after receiving a complete nutrition drink fortified with EPA + BCAA 2:1:1 and 4:1:1). This favorable effect may be explained by the combinatorial mechanisms of promoting muscle protein synthesis and prevention of muscle loss from inflammation. In this group, plasma EPA and BCAA levels were also increased significantly along with the tendency in increasing muscle protein synthesis marker - carnosine and anti-inflammatory marker - IL-10 and decrease muscle degradation marker - histidine and pro-inflammatory marker - IL-6.

Sarcopenia occurs through age-related deterioration in muscle protein synthesis along with chronic inflammation accelerating the process of muscle protein degradation (34). While pro-inflammatory cytokines such as IL-6, IL-1, and TNF-α induce inflammation and promote muscle degradation, anti-inflammatory cytokines such as IL-10 inhibit inflammation and prevent sarcopenia (35). Numerous studies have shown the association between elevated pro-inflammatory markers and reduced muscle mass and strength in the sarcopenic elderly (36). Using a combination between EPA and BCAA, we found a non-significant trend in the decrease of pro-inflammatory cytokines and an increase in anti-inflammatory cytokines, which is likely derived from the effect of EPA. A reduced inflammatory state may result in decrease muscle degradation as we observed a tendency in the decrease of free amino acid histidine after interventions. Furthermore, the tendency to increase muscle protein synthesis marker carnosine likely stems from the effect of BCAA. Taken together, the findings from this short-term study suggest that the combination of EPA and BCAA might have the potential to reduce inflammatory-related muscle protein degradation and increase muscle protein synthesis. Future long-term studies are warranted to investigate the anti-inflammatory and muscle synthesis-promoting effect of this EPA-BCAA combination. More inflammatory biomarkers including PAF and TNF-α should also be measured besides the interleukins. It is worth noting that the significant effect was observed only in the right arm but not the left arm or total body muscle mass. The reason for this finding may be explained by the arm exercise instructed in this study. Though we asked all participants to do the exercise in both arms, most participants are right-handed. Future studies are warranted to investigate further if full-body exercise could help improve total muscle mass.

A high BCAA and/or leucine content is generally required for stimulating protein synthesis in skeletal muscle tissue (37). Generally, amino acids serve as a substrate, but branch-chain amino acids especially leucine can directly activate muscle protein synthesis by activating the mechanistic target of rapamycin (mTOR) signaling (38, 39). Leucine was essential for triggering the mammalian target of rapamycin complex 1 (mTORC1), as well as the downstream phosphorylation of p70S6 kinase (p70S6k) and 4E (eIF4E)-binding protein 1 (4E-BP1) and related signaling pathways in muscle rejuvenation (38, 39). Interestingly, in the current study, we also found more favorable effects in the group receiving EPA + BCAA (4:1:1 of Leu: Ile: Val) than that of EPA + BCAA (2:1:1 of Leu: Ile: Val), suggesting the importance of leucine. However, the previous study highlighted that BCAA intake had to be consistent to successfully maintain muscle mass (33). According to repeated measures for plasma BCAA changes, we also observed significantly elevated plasma leucine and isoleucine in the group with EPA + BCAA (4:1:1). These outcomes confirm that the intervention really cause an increase in the bioavailable amino acids. Consistently, our finding suggests that supplementation with EPA + BCAA 2:1:1 and 4:1:1 for 3 weeks may improve right arm muscle mass and strength. However, the changes are still not significantly different from the placebo or EPA only. It is worth noting that in this study, we also observed the elevation of plasma BCAA after consuming the unfortified complete nutrition drink (placebo) and the one fortified with EPA only. The results may be because the complete nutrition drink can provide both energy and protein, which could be digested further to BCAA. The elevation of plasma BCAA in the placebo or EPA only may explain the non-statistically significant of right arm muscle mass and strength among different groups. Future studies with longer duration of intervention are warranted to investigate the effect of the EPA + BCAA combination.

A previous meta-analysis of 10 randomized control trials showed that omega-3 fatty acid supplements at more than 2 g/day may contribute to muscle mass gain (0.67 kg; 95% CI: 0.16, 1.18) and improve walking speed (1.78 m/sec; 95% CI: 1.38, 2.17), especially for those receiving more than 6 months of intervention (40). For example, Smith et al. showed that daily consumption of fish oil for 6 months resulted in improved muscle mass, handgrip strength, and muscle performance (41). In the current study of 3 weeks, combining 2 g/ day of EPA with 5 g/ day of BCAA in a complete nutrition drink plus hand exercise showed a significant increase in right arm muscle mass and strength along with non-significant elevation in muscle protein synthesis marker. The findings suggest that the combination may help facilitate the efficacy of a short-term intervention. A more recent meta-analysis found that omega-3 fatty acid supplements did not affect muscle mass but improved muscle strength and muscle performance in older adults (42). The potential mechanisms of omega-3 fatty acids to promote muscle mass and physical performance include anti-inflammatory effects, mTOR pathway, and reduction of insulin resistance (43). Its anti-inflammatory effect is most well-studied. In fact, a meta-analysis study confirmed that there was a reduction in inflammation markers (i.e., CRP and IL-6) after taking supplementation with omega-3 PUFAs in middle-aged and older adults (44).

Our current study shows the tendency of decreased IL-6 and increased IL-10 plasma levels after consuming fortified formula with EPA and EPA+BCAA (4:1:1). While the mechanisms underlying sarcopenia remained to be elucidated, chronic low-grade inflammation is one of the most documented mechanisms of sarcopenia. The elevations in cytokines (i.e., IL-6 and TNF-α) were found to correlate with functional disability and may be involved in sarcopenia through effects on pathways controlling protein metabolism (43). In this study, we found a significant elevated plasma EPA after consuming the complete nutrition drink with 2.2g EPA only, and the 2.2g EPA with BCAA (4:1:1). For comparison among groups, a significantly elevated plasma EPA was found in the non-hydrolyzed form in the group consuming 2.2g EPA with BCAA (4:1:1) compared to the placebo control (p=0.0003). The findings suggest that the addition of EPA in complete nutrition drinks could be a more promising available source of EPA than a regular diet with iso-caloric control drinks. In recent years, the n-3 PUFAs found in fish oil were studied to treat sarcopenia as an anti-inflammation related to the maintenance of muscle health in older adults (45). Primarily, a former study in community-dwelling elderly found that n-3 fatty acid intake was 19% different among elderly that were diagnosed with sarcopenia and non-sarcopenia (*p* = 0.005) (46). In 2017, a remarkable randomized control trial investigated the effects of EPA and DHA therapy on inflammation in older adults. Supplementation had a significant decreasing effect on IL-6, IL-1β, and TNFα levels after 4 weeks of use and was even greater after 8 weeks (47). An *in vitro* study in a mouse model highlighted novel pathways associated with lipotoxicity and cytotoxicity in the potential targeting of molecular modulators of sarcopenic obesity, with mice fed with EPA-rich food under cytotoxic stress (TNF-α) shown to partially rescue differentiation with enhanced myotube formation of mouse skeletal tissue (48). In our current study, although the inflammation markers did not express significant changes, a tendency of decreased pro-inflammatory cytokine such as IL-6 and an increased anti-inflammatory cytokine such as IL-10 was found, suggesting a potential reduction in inflammation. Future studies with longer duration of treatment may be required to see the significant anti-inflammatory effect of EPA in the elderly at the risk of sarcopenia.

Monitoring of muscle turnover with carnosine, histidine, and β-alanine was applied to measure the rate of protein breakdown. A tendency of increased carnosine and decreased histidine levels was found after consuming fortified formula with EPA and BCAA (4:1:1), compared to other groups. An increase in dipeptide (carnosine) and a decrease in free amino acid (histidine) suggest a shift toward the anabolism of muscle protein. This finding is consistent with the observed effect on promoting muscle strength and muscle mass. Carnosine (β-alanyl-L-histidine) is an intramuscular dipeptide consisting of β-alanine and L-histidine (49). Therefore, the changes in the ratio between the free amino acids (β-alanine and histidine) and the dipeptide carnosine can be an indicator of changes in muscle protein synthesis and degradation. Moreover, a plasma lipid profile of lower n6, which antagonize n3, has been associated with reduced PAF biosynthesis and/or increased catabolism (21). However, this study did not find significant differences among groups of treatment. Future studies with a longer duration of consuming a fortified formula with EPA and BCAA (4:1:1) are warranted.

The strength of this study is the randomized placebo design with the comparison between EPA alone, EPA + BCAA at ratios 2:1:1 and 4:1:1 of Leu: Ile: Val. Furthermore, a significant increase in EPA, leucine, and isoleucine in the bloodstream assures effective supplementation and compliance. Compared to other studies, this study utilized a much shorter duration of treatment with BCAA but still can show some tendency of positive changes in all important parameters. Nevertheless, short-term supplementation (3 weeks) and small sample size are limitations for this study to see a statistical difference between groups. Therefore, a longer period of the clinical trial in a larger sample size may be needed to investigate the effect of the EPA-BCAA combination. It was worth noting that the results of this study were summarized by the average values from all participants comparing the consequences of EPA and BCAA among groups of intervention. The actual results for each individual were varied. Future studies should be performed to characterize the responders and non-responders according to their background such as genetic polymorphisms. In future, we hope that the specific fortified complete nutrition drinks may be used for the precise person as the concept of personalized nutrition.

## Conclusion

This 3-weeks clinical trial demonstrated that the EPA or EPA–BCAA combination fortified complete nutrition drinks were all well-tolerated. A significant improvement in right arm muscle mass and right handgrip strength was found after receiving a complete nutrition drink fortified with EPA and BCAA 2:1:1 and 4:1:1 of Leu: Ile: Val. However, the changes are not statistically different from those of control or EPA-only formulas. The plasma metabolite of EPA, leucine, and isoleucine was significantly increased among all interventions within 3 weeks. No significant changes in inflammatory cytokines and muscle degradation markers were observed. Nevertheless, the tendency of decreased inflammatory cytokines and increased inflammatory cytokines, decreased histidine, and increased carnosine was observed after consuming EPA and BCAA (4:1:1) compared to the placebo control group. This clinical trial suggested that the fortification of EPA together with a high proportion of leucine in BCAA (4:1:1) formula elevated available plasma EPA and BCAA. These findings suggest that consuming a complete nutrition drink fortified with EPA and BCAA 2:1:1 and 4:1:1 for 3 weeks might improve right arm muscle mass and strength likely by the tendency to promote muscle protein synthesis and anti-inflammatory effects. Future studies with longer duration are warranted to confirm it.

## Data availability statement

The original contributions presented in the study are included in the article/Supplementary material, further inquiries can be directed to the corresponding author.

## Ethics statement

The studies involving human participants were reviewed and approved by the Mahidol University Central Institutional Review Board (MU-CIRB), Mahidol University, Thailand. The patients/participants provided their written informed consent to participate in this study.

## Author contributions

WK: designed the manuscript, obtained ethical approval, collected data, performed laboratory analyses, statistical analyses, and drafted the manuscript. PS and CS: designed the manuscript and provided scientific input in the discussion of the data. KP: collected data. DT: obtained the grant, designed the manuscript, performed randomization, supervised laboratory analysis, and edited the manuscript. All authors contributed to the article and approved the submitted version.
