# Supplementary material for: Eicosapentaenoic acid and branched-chain amino acids fortified complete nutrition drink improved muscle strength in older individuals with inadequate protein intake
Source: Front Nutr. 2023 Jun 30;10:1164469. doi: 10.3389/fnut.2023.1164469 (PMC10349202; doi:10.3389/fnut.2023.1164469)
Supplement: Supplementary file 1 [file Data_Sheet_1.PDF]

Figure S1: conceptual framework

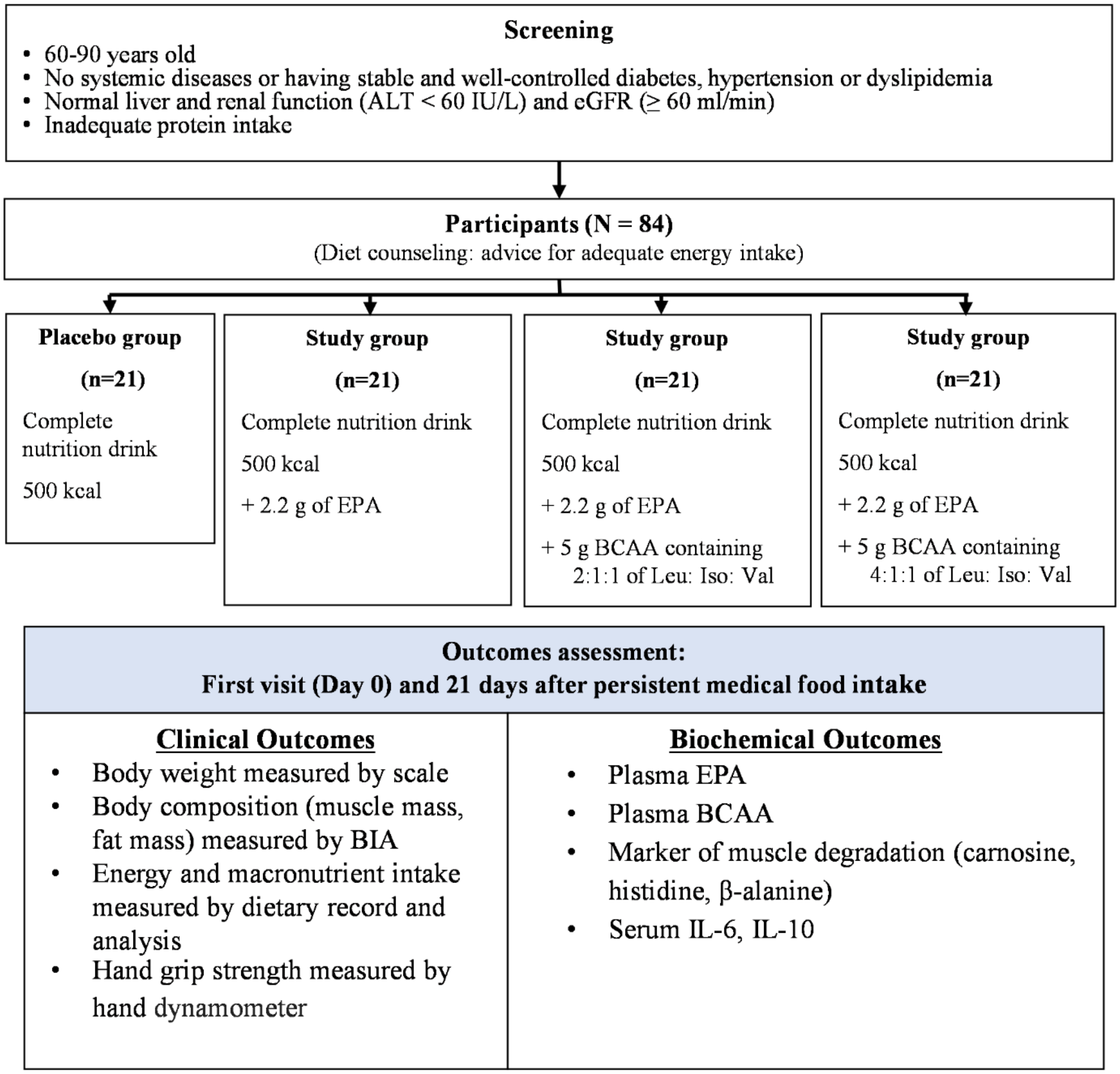

Table S1: Comparison of ingredients in all four formulas of complete nutrition drinks

Ingredient	Formula 1 Control (g/250ml serving)	Formula 2 EPA (g/250ml serving)	Formula 3 EPA+BCAA (2:1:1) (g/250ml serving)	Formula 4 EPA+BCAA (4:1:1) (g/250ml serving)
Maltodextrin	22.14	22.14	22.14	22.14
Sucrose	11.00	11.00	11.00	11.00
Soy protein	9.37	9.37	9.37	9.37
Rice bran oil	7.61	7.61	7.61	7.61
Multi mineral mixture	1.25	1.25	1.25	1.25
Fructo-Oligosacharide (FOS)	1.24	1.24	1.24	1.24
Sodium caseinate	0.81	0.81	0.81	0.81
MCT oil	0.66	0.66	0.66	0.66
Multi vitamin mixture	0.19	0.19	0.19	0.19
EPA	-	1.10	1.10	1.10
Leucine	-	-	1.25	1.66
Isoleucine	-	-	0.62	0.41
Valine	-	-	0.62	0.41

Table S2: Gradient condition for analysis of plasma carnosine, β-alanine, and histidine via LC-MS/MS

No	Time	Flow [ml/min]	%B	%C	%D	Curve
1	0.000	Run				
2	0.000	0.200	3.0	0.0	0.0	5
3	0.890	0.200	10.0	0.0	0.0	5
4	2.670	0.200	50.0	0.0	0.0	5
5	4.440	0.200	90.0	0.0	0.0	5
6	5.000	0.200	3.0	0.0	0.0	5
7	*New Row*					
8	5.000	Stop Run				

Table S3: Comparison of energy and protein intake calculated from the dietary record and estimated into average nutrition intake per day

Intakes	Group 1 Control			Group 2 EPA			Group 3 EPA+BCAA (2:1:1)			Group 4 EPA+BCAA (4:1:1)		
	**Baseline**	**3 weeks**	**p-value**	**Baseline**	**3 weeks**	**p-value**	**Baseline**	**3 weeks**	**p-value**	**Baseline**	**3 weeks**	**p-value**
Average energy intake	1108 ± 411.7	1489 ± 531.1	*	1300 ± 318.9	1709 ± 294.2	*	1190 ± 189.3	1608 ± 190.5	*	1207 ± 446.7	1591 ± 510.8	*
Average protein intake	50.91 ± 21.26	66.98 ± 25.85	0.1	55.03 ± 12.45	72.33 ± 11.37	0.07	50.39 ± 16.27	68.03 ± 15.34	0.06	53.24 ± 21.79	69.50 ± 24.12	0.11
Average carbohydrate intake	138.3 ± 55.17	188.2 ± 70.44	0.1	178.8 ± 56.63	232.50 ± 55.00	0.08	152.1 ± 49.20	206.90 ± 49.51	0.07	158.50 ± 67.97	209.10 ± 75.39	0.13
Average fat intake	39.09 ± 16.60	52.00 ± 20.06	0.1	40.51 ± 12.49	54.40 ± 11.35	0.06	36.99 ± 13.59	51.16 ± 12.73	0.05	39.94 ± 14.89	53.00 ± 17.11	0.10

Data were expressed as mean ± standard deviation (SD). The statistical differences between baseline and three weeks after intervention for each group were analyzed by using paired t-tests.
* represents $p < 0.05$.

Table S4 Changes of blood biochemistry before and after receiving medical food of each group following with assigned hand grip practice for 3 weeks within each group

Parameters	Group 1 Control			Group 2 EPA			Group 3 EPA+BCAA (2:1:1)			Group 4 EPA+BCAA (4:1:1)		
	Visit 1	Visit 2	p-value	Visit 1	Visit 2	p-value	Visit 1	Visit 2	p-value	Visit 1	Visit 2	p-value
Glu-cose	103.50 ± 9.57	95.10 ± 11.04	0.3604	100.50 ± 7.79	95.67 ± 7.96	0.8944	101.60 ± 7.19	93.40 ± 9.19	0.3926	106.90 ± 16.02	101.60 ± 21.40	0.8727
Hb A1C	5.69 ±0.41	5.69 ±0.39	>0.9999	5.40 ±1.28	5.38 ±1.28	>0.9999	5.54 ±0.36	5.54 ±0.35	>0.9999	5.88 ±1.20	5.55 ±1.82	0.9774
Tri glyce ride	134.90 ± 72.93	129.10 ± 61.75	>0.9999	105.90 ± 30.14	100.50 ± 28.24	>0.9999	108.90 ± 30.38	100.60 ± 33.58	0.9996	127.70 ± 69.50	117.30 ± 60.21	0.9984
Chole sterol	196.80 ± 29.13	192.20 ± 31.34	0.9999	218.00 ± 22.84	203.70 ± 28.69	0.8679	221.50 ± 42.21	206.40 ± 26.75	0.8461	216.20 ± 42.44	198.60 ± 40.82	0.7448
HDL	59.10 ±10.26	60.00 ±13.15	>0.9999	61.14 ±10.51	62.76 ±12.43	0.9998	62.65 ±8.00	67.25 ±13.35	0.9118	63.68 ±10.29	62.58 ±13.23	>0.9999
LDL	110.70 ± 24.02	106.40 ± 29.62	0.9999	136.30 ± 24.80	120.80 ± 29.02	0.7809	137.10 ± 43.23	119.00 ± 28.70	0.6414	126.90 ± 39.22	113.70 ± 36.71	0.9151

Data were expressed as mean ± standard deviation (SD). The statistical differences between visit 1 (baseline) and visit 2 (three weeks after intervention) for each group were analyzed using paired t-tests.
